# A Digital Improvement—Trimming a Digital Temperature Sensor with EEPROM Reprogrammable Fuses

**DOI:** 10.3390/s21051700

**Published:** 2021-03-02

**Authors:** Anca Mihaela Vasile (Dragan), Alina Negut, Adrian Tache, Gheorghe Brezeanu

**Affiliations:** 1 ON Semiconductor Romania, 020983 Bucharest, Romania; Alina.Negut@onsemi.com (A.N.); AdrianMacarie.Tache@onsemi.com (A.T.); 2 Faculty of Electronics, Politehnica University of Bucharest, Telecommunications and Information Technology, 999032 Bucharest, Romania; gheorghe.brezeanu@dce.pub.ro

**Keywords:** integrated circuits, EEPROM reprogrammable fuses, memory cells, trimming techniques with fuses, digital temperature sensor, temperature sensor with digital serial interface

## Abstract

An EEPROM (electrically erasable programmable read-only memory) reprogrammable fuse for trimming a digital temperature sensor is designed in a 0.18-µm CMOS EEPROM. The fuse uses EEPROM memory cells, which allow multiple programming cycles by modifying the stored data on the digital trim codes applied to the thermal sensor. By reprogramming the fuse, the temperature sensor can be adjusted with an increased trim variation in order to achieve higher accuracy. Experimental results for the trimmed digital sensor showed a +1.5/−1.0 ℃ inaccuracy in the temperature range of −20 to 125 ℃ for 25 trimmed DTS samples at 1.8 V by one-point calibration. Furthermore, an average mean of 0.40 ℃ and a standard deviation of 0.70 ℃ temperature error were obtained in the same temperature range for power supply voltages from 1.7 to 1.9 V. Thus, the digital sensor exhibits similar performances for the entire power supply range of 1.7 to 3.6 V.

## 1. Introduction

Digital temperature sensors are suitable for thermal and power management systems of PCs, laptops, and smartphones. A low-cost and high-accuracy temperature sensor with a digital interface is desired nowadays due to the high demand for electronic smart gadgets [1,2,3,4]. Furthermore, the use of temperature detectors with digital output has been recently reported in smart sensor networks, image sensors, Internet of Things devices, and biomedical applications [5,6,7,8,9].

Modern CMOS smart temperature sensors are categorized according to the sensing device (BJT, MOS in subthreshold region and resistor) or the physical principle on which the temperature is detected (bandgap voltage and thermal diffusion (TD)) [7,8]. Increased accuracy, high precision, and output linearity with low power consumption are some of the most important targets to achieve when designing such integrated sensors [10,11,12,13,14,15,16,17,18,19,20]. The aim to meet these requirements with low production costs becomes more and more challenging these days. The limitation for achieving higher accuracy for a thermal sensor is based on a trade-off between the production costs for calibration and the required precision [21].

In deep submicron processes, nonideal factors like the temperature coefficient of the devices, components mismatch, or absolute deviation of resistance affect the performance of a smart sensor. Furthermore, process spread and packaging stress play important roles in limiting the accuracy of a thermal sensor [22,23,24]. Thus, calibration methods and trimming techniques are required to achieve the imposed performances of a digital temperature sensor.

A calibration procedure provides information about the accuracy of a thermal sensor [22,23,24,25,26]. Most circuits are calibrated to two well-defined temperature points, after which the circuit is adjusted to minimize the temperature error by trimming techniques [23]. A one-point calibration has the advantage of lower production costs [25].

Smart sensors are usually calibrated by comparing them with a reference thermometer of known accuracy. The calibration can be done at the wafer level or after packaging. Regarding wafer-level calibration, the temperature of a complete wafer is stabilized and measured using a number of reference thermometers mounted on the wafer chuck [26]. Several electrical tests and temperature readings from the chip are performed, followed by adjustments in order to meet its performance requirements. Calibration after packaging implies achieving the same temperature for every individually packaged IC (integrated circuit) as for the reference thermometer in a thermally conducting medium, such as a liquid bath or a metal block [26]. 

After calibration at wafer level or after packaging, smart temperature sensors usually require an adjustment for the targeted parameter by applying a digital trim code [27]. Conventional methods consist of a permanent modification of the IC by laser trimming or by altering metal fuse links [28]. Nowadays, a one-time programmable fuse (OTP fuse) is often used for trimming a thermal sensor due to its ability to store the values of the trim codes in a data latch and, for instance, to allow two states for the digital trim code [23,27,29]. 

For any of these trimming techniques, once the fuse is trimmed, it cannot return to its original state [1]. Thus, a more complex trim involves several programming cycles. Furthermore, trimming an integrated sensor with OTP fuses requires a lot of extra pads, which are not accessible to the user, in order to store the digital trim codes required for calibration [27]. Using EEPROM memory cells (EEcells), the fuse can be reprogrammed, allowing multiple programming cycles for trimming the digital temperature sensor. The endurance of an EEPROM memory cell, without altering its precision in time, covers around 1,000,000 programming cycles, while its data retention exceeds 100 years [2]. With this technique, the thermal sensor can be trimmed in an increased trim variation with multiple digital codes, offering an efficient way to achieve its performance requirements. For instance, the benefit of using the proposed EEPROM technique is that it allows an increased number of digital trim codes for calibrating the circuit, with a low production cost.

In comparison with OTP fuses, an EEPROM fuse offers multiple advantages, such as an increased number of programming cycles and no extra pads required for trimming, which reduce the area consumption of the IC. Furthermore, the reprogrammable fuse allows retrimming when the IC’s specifications are changed by the beneficiary. Additionally, testing/trimming time is drastically reduced, resulting in a lower production cost. Moreover, an important advantage of using EEPROM fuses includes the possibility of choosing the number of fuses used to achieve the desired accuracy of the thermal sensor. 

In this paper, a digital trimming technique with reprogrammable fuses for a digital temperature sensor (DTS) is proposed. The fuse uses EEPROM memory cells, which allow multiple programming cycles by altering the stored data of the digital trim codes. Thus, the digital sensor can be adjusted with an increased trim variation in order to achieve higher accuracy. The thermal sensor with the digital trim is designed and implemented in a 0.18-µm CMOS EEPROM process.

## 2. EEPROM Reprogrammable Fuse

**Figure 1:**
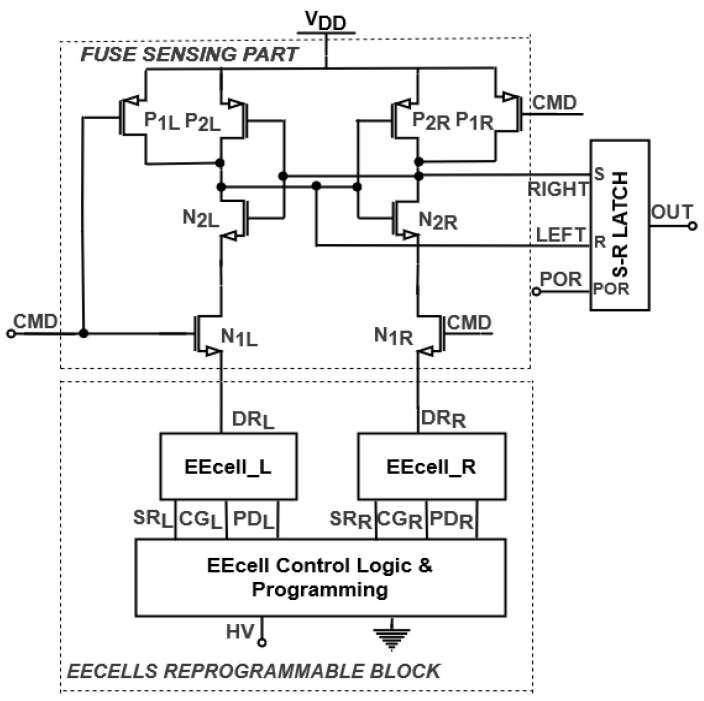
Schematic of the EEPROM reprogrammable fuse [1].

An EEPROM reprogrammable fuse is proposed in Figure 1 [1]. The circuit includes a fuse-sensing part formed by two controlled current paths, *LEFT* and *RIGHT*, with four switching transistors, an output S-R latch, and two EEPROM memory cells, *EEcell_L* and *EEcell_R*, and an EEcell Control Logic & Programming Block [1]. The state of the fuse is controlled by the command signal *CMD*, while a programming signal is provided by the EEcell Control Logic & Programming Block. 

EEcells have electrically isolated gates, storing data in the form of a charge on the floating gate (FG). The charge is transported to the FGs in the programming operation. The EEcells have four terminals: drain read (*DR*), control gate (*CG*), source read (*SR*), and programmable drain (*PD*). 

By appropriate programming controlled by the EEcell Control Logic & Programming Block, a high voltage (HV) is applied to the control gate or the programmable drain terminal. When programming the EEcells with “1”, the FG potential has a positive value, determining *DR* to provide a path to ground. In the scenario of “0”, a negative value is stored on the floating gate, switching the *DR* signal to high, while the path to ground is disconnected. In order to maintain proper operation of the fuse-sensing part, *EEcell_L* and *EEcell_R* are programmed with complementary data.

**Figure 2:**
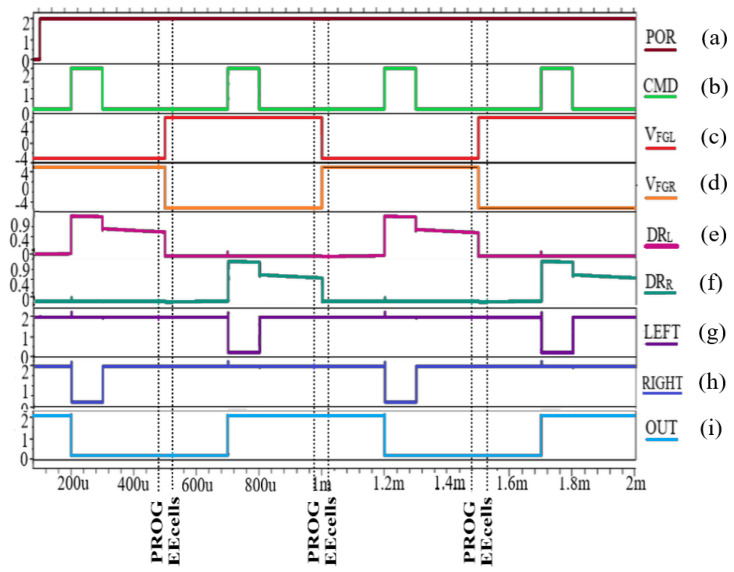
Detailed EEPROM reprogrammable fuse waveforms: (**a**) *POR*, (**b**) *CMD*, (**c**) *V_FGL_*, (**d**) *V_FGR_*, (**e**) *DR_L_*, (**f**) *DR_R_*_,_ (**g**) *LEFT*, (**h**) *RIGHT*, (**i**) *OUT* [1].

The novel fuse presented in Figure 1 was designed using a 0.18-µm CMOS EEPROM process with low voltage (2V) transistors, while its operation was tested through Synopsys HSPICE^®^ (HSPICE is a trademark of Synopsys Inc. in the US and/or other countries.) simulations, with the resulting waveforms shown in Figure 2. The circuit is supplied at power supply voltage *V_DD_* = 2 V, while for programming the EEcells, a high voltage (HV) is applied to the EEPROM memory cells.

After power-up and initialization of the output S-R latch, *POR* switches from “0” to logic “1” (Figure 2a). When *CMD* is asserted (*CMD* = 1; Figure 2b), the fuse enters evaluation mode. In the first scenario, *EEcell_L* is programmed with “0” and a negative value is stored on the FG (*V_FGL_* = −4 V; Figure 2c), while *EEcell_R* is programmed with “1” (*V_FGR_* = 4 V; Figure 2d). In this case, the current path to ground is provided by the right branch (*DR_R_* = 0; Figure 2f), pulling *RIGHT* to “0” (Figure 2h), while *LEFT* is tied to *V_DD_* (Figure 2g). Thus, the output latch stores “0”, while the output of the fuse will be in “0” logic (Figure 2i) [1].

The second EEcell programming is done with complementary data. When the fuse is evaluated again (Figure 2b), a positive value is stored on *FG_L* (*V_FGL_* = 4 V; Figure 2c), and the floating gate potential of *EEcell_R* has a negative value (*V_FGR_* = −4 V; Figure 2d). At this time, *DR_L_* is pulled to ground (Figure 2e), while *DR_R_* switches to 0.9V (Figure 2f). Thus, *LEFT* is pulled to ground (Figure 2g), while the *RIGHT* signal will be in logic “1” (Figure 2h). For this scenario, the S-R latch will memorize “1” logic (OUT = 1; Figure 2i) [1]. 

Two similar scenarios for reprogramming the fuse are represented in Figure 2, showing the capability to change the fuse multiple times by reprogramming the EEcells. This provides the advantage of being able to trim a smart sensor with various digital trim codes until the desired parameters are obtained. Thus, the proposed fuse is used for trimming a digital temperature sensor in order for it to exhibit increased accuracy. 

**Figure 3:**
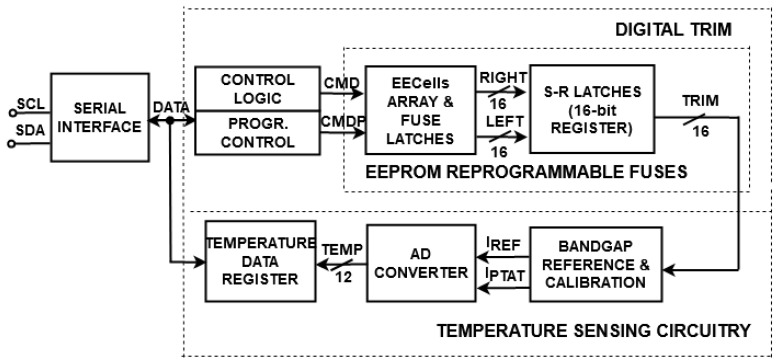
Block schematic of digital temperature sensor (DTS) [1].

In addition to the benefit of being able to reprogram the fuse, the proposed trimming technique achieves lower area consumption compared to a metal fuse implementation. The digital trim of the temperature sensor presented in Figure 3, which includes 16 proposed EEPROM fuses, occupies only 0.030067 mm^2^ of the total chip area of 2.07 mm^2^. A metal fuse trimming technique for another temperature sensor implementation, which contains 7 trim pads, occupies 0.05077 mm^2^ of the 0.3195 mm^2^ chip area, representing almost ¼ of total area consumption. Thus, the proposed fuse technique has twice-lower area consumption than the metal fuse implementation and allows more digital trim codes that can be reprogrammed.

## 3. Trimming a Digital Temperature Sensor with EEPROM Fuses 

A digital temperature sensor (DTS) with the proposed EEPROM reprogrammable fuse is shown in Figure 3. The smart sensor includes a serial interface, a digital trim block, and temperature sensing circuitry. The interface communicates with the trimming circuitry in order to provide the digital trim codes required for adjusting temperature sensing. Furthermore, after detecting the temperature in a digital representation, the sensing circuitry communicates with the interface by sending the data stored in a temperature data register [30].

The interface has two serial communication lines: a serial clock line, *SCL*, which is an input pin, and a serial data line, *SDA*, a bidirectional pin. The serial interface sends the data for controlling 16 EEPROM reprogrammable fuses (Figure 1) with a *CMD* signal (for evaluating the fuse) and a *CMDP* signal (for programming the EEcells) [1]. Accordingly, with the programmed EEcells (Figure 2), the trimming circuitry offers digital trim codes from 0 × 0000 (minTRIM) to 0 × FFFF (maxTRIM), allowing the digital sensor to be adjusted in order to achieve increased accuracy [1].

The core of the temperature-sensing circuitry is a bandgap reference with calibration, which provides a PTAT current (*I_PTAT_*), and a reference current (*I_REF_*). The AD converter (analog to digital converter) compares the analog values and offers the digital representation of the temperature (*TEMP*). The digital value is stored in the temperature data register, which is sent to the serial interface.

**Figure 4:**
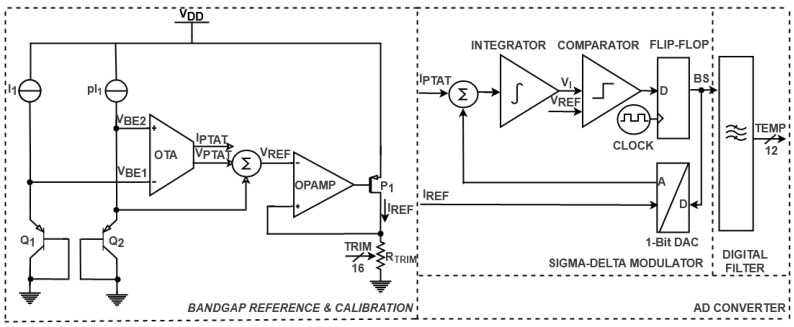
Detailed temperature sensing circuitry of DTS.

A detailed view of the temperature-sensing circuitry of the DTS is illustrated in Figure 4. The bandgap reference and calibration include two identical BJT transistors, *Q*_1_ and *Q*_2_, which are biased with *I_1_* and *pI_1_* currents [26]. The positive to absolute temperature values (*V_PTAT_*, *I_PTAT_*) are obtained by subtracting the base-emitter voltages of the sensing devices (*V_BE1_*, *V_BE2_*) [26]. The reference voltage (*V_REF_*) is expressed as the sum between the resulting positive voltage *V_PTAT_* and the base-emitter voltage *V_BE2_.* In order to acquire the reference current (*I_REF_*), a buffer (*OPAMP*) and the resistor (*R_TRIM_*) are used [31]. The bandgap reference and calibration are trimmed by adjusting *I_RE_*_F_ in order to improve the accuracy of the digital temperature sensor.

The AD converter of the DTS described in Figure 4 comprises a sigma-delta modulator and a digital filter. The sigma-delta modulator processes the analog currents *I_PTAT_* and *I_REF_* and generates a bit stream signal (BS) [32]. The integrator of the sigma-delta modulator stores the difference between the analog currents *I_PTAT_* and *I_REF_*, while the rising voltage *V_I_* is compared with *V_REF_*. The output of the comparator is then sampled by a clocked flip flop, which synchronizes the received data with the clock. The resulting signal (*BS*) is fed back into the system by a 1-bit DAC converter, which acts as a switch for the loop. When BS is in logic “0”, only *I_PTAT_* will be processed by the integrator, while in logic “1”, the difference between the analog values will be taken into account. The output of the sigma-delta modulator is then processed through the digital filter, which generates a filtered multibit digital signal by oversampling and decimation techniques [33,34]. Thus, the temperature detected by the BJT transistors is represented in a digital format (*TEMP* [0:11]).

**Figure 5:**
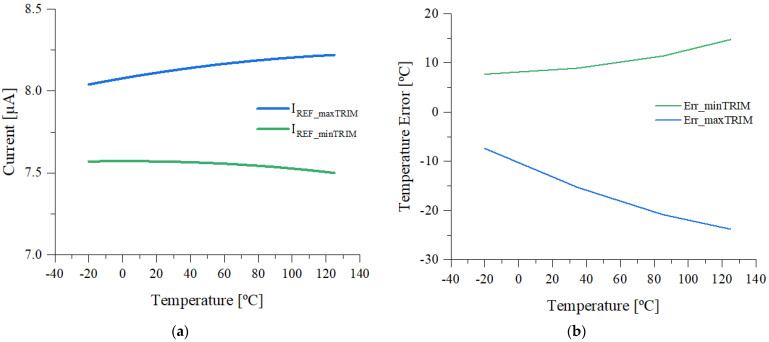
(**a**) Simulated dependence of the *I_REF_* with trim variation and temperature for DTS (**b**) simulated temperature error of DTS with trim variation.

The digital temperature sensor depicted in Figure 3 was designed using a 0.18-µm CMOS EEPROM process. The system operates at supply voltages from 1.7 to 3.6 V. The operation of the trimmed DTS was observed through Synopsys HSPICE simulations and wafer-level and encapsulated IC measurements. A DC sweep analysis for the trimmed current *I_REF_* in the temperature range of −20 to 125 °C, with minTRIM and maxTRIM digital codes, is depicted in Figure 5a. The reference current can be adjusted between 7.5 and 8.5 µA, depending on the digital trim code.

The temperature error of the DTS applied to *I_REF_* is shown in Figure 5b. A negative slope of the error vs. temperature variation results from trimming *I_REF_* with maxTRIM, while a positive slope is observed by applying minTRIM. By modifying the slope of the temperature error through the trim applied to *I_REF_*, an increased accuracy can be achieved. Thus, an optimal digital trim code is available for the reference current, which provides a minimum temperature error.

**Figure 6:**
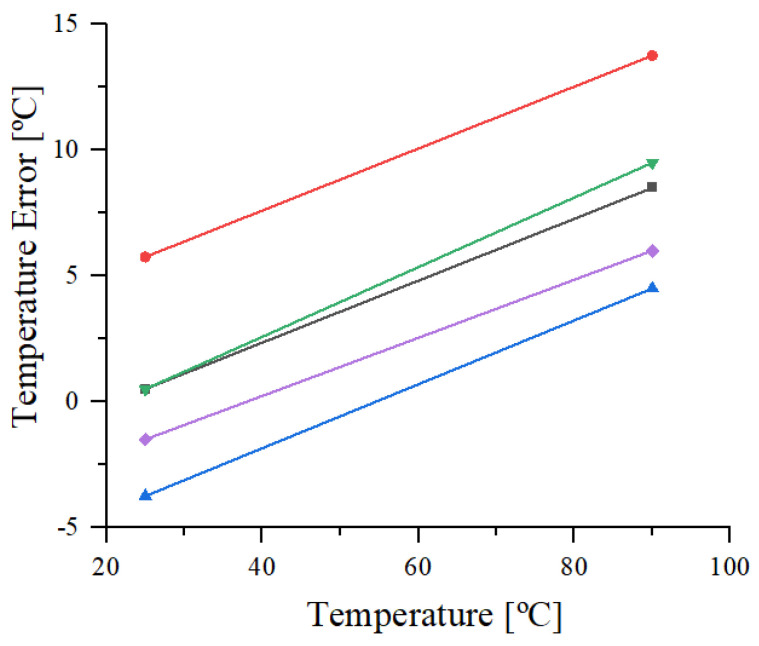
Measured temperature error of 5 untrimmed DTS samples at 1.8 V.

The DTS’s measurements were performed at the wafer level and on ceramic encapsulated ICs. For testing the untrimmed sensor’s performances, wafer-level measurements were carried out at room and hot temperatures (25 and 85 ℃). The temperature error for 5 measured untrimmed DST samples is represented in Figure 6. For half of the samples, a variation of −2 ℃ inaccuracy can be observed for both measured temperatures, while a maximum −9.5 ℃ variation of temperature error is obtained. Furthermore, the investigated DTS samples show a +13.75/−5 ℃ inaccuracy for the untrimmed DTS.

**Figure 7:**
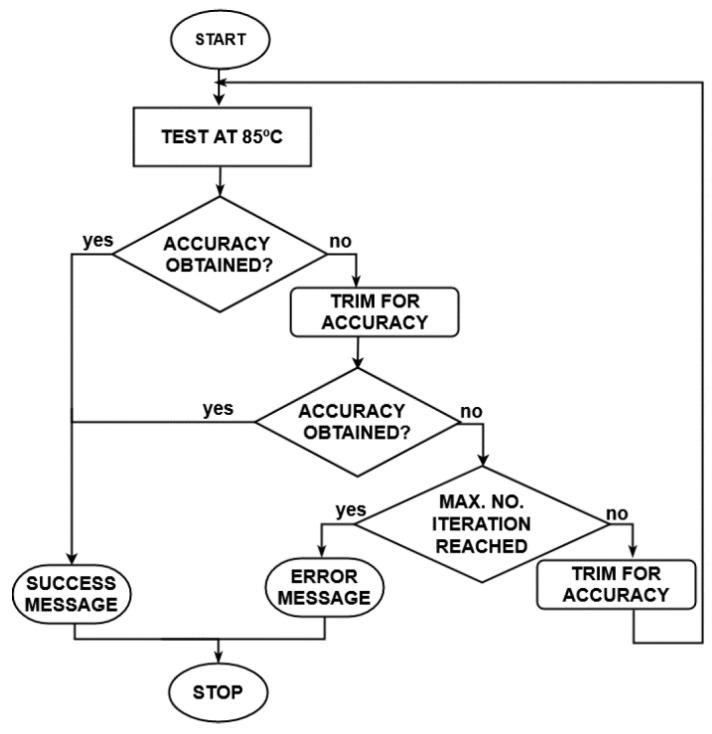
One-point calibration diagram.

In order to evaluate the trimmed sensor’s inaccuracy for the entire temperature range, measurements on encapsulated ICs were investigated. The test setup involves the test system Maverick-II and the micro-bath calibrator Fluke7103 [35], which control the environmental temperature precisely. The one-point calibration method follows the steps described in Figure 7. The circuit is calibrated at 85 ℃. If accuracy is achieved, the process is finished and the optimum digital trim code is found. Otherwise, the investigations continue until accuracy is obtained or the trim variation reaches all its digital trim codes.

**Figure 8:**
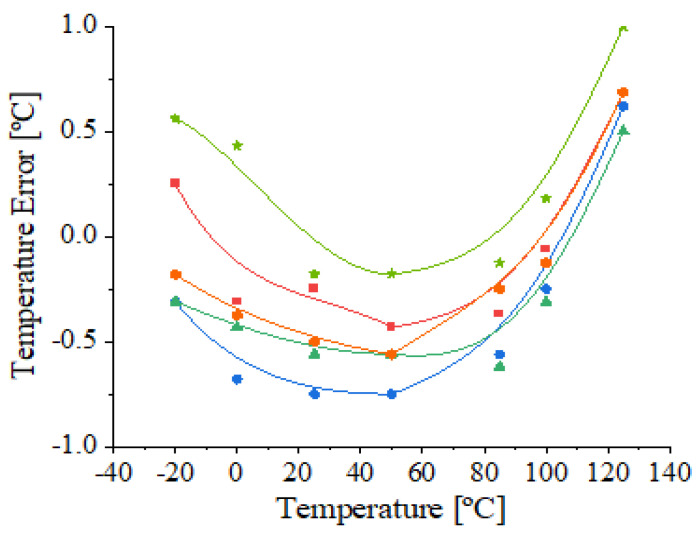
Measured temperature error of 5 trimmed DTS samples at 1.8 V.

*I_REF_* is adjusted with the optimal digital trim code during the test process for each fabricated sample. DTS samples (supplied at 1.8 V) with the optimal trim code were measured in the temperature domain of −20 to 125 ℃. The temperature error of the DTS for 5 encapsulated ICs is illustrated in Figure 8. A fairly low inaccuracy of +1/−0.75 ℃ was obtained. Furthermore, for most of the samples, the temperature error varied by ±0.5 ℃ for the entire temperature range. Thus, a 0.24 ℃ average mean with a 0.44 ℃ standard deviation of the measured error was obtained for the full temperature range. The advantage of using this proposed digital trim is the possibility of adjusting each sample with an optimal digital trim code in order to minimize the effect of device spread for a given technology. Additionally, in comparison with the measurements of the untrimmed sensor depicted in Figure 6, the inaccuracy illustrated in Figure 8 is more than 5 times lower for the entire investigated temperature range. 

**Figure 9:**
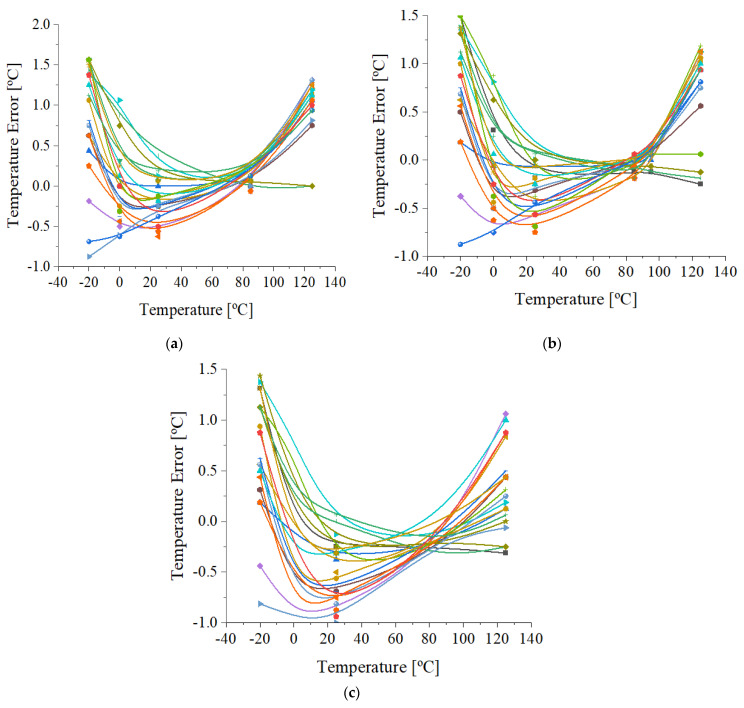
(**a**) Measured temperature error of 20 trimmed DTS samples at 1.7 V; (**b**) measured temperature error of 20 trimmed DTS samples at 1.8 V; (**c**) measured temperature error of 20 trimmed DTS samples at 1.9 V.

Further investigations of the digital temperature sensor’s inaccuracy were performed by measuring 20 DTS samples. The measured error dependence as a function of temperature for three power supply values is presented in Figure 9a–c. The full DTS temperature range was tested. The means and standard deviations of the temperature errors for the measured samples at each supply voltage are shown in Table 1.

The measured error data of samples supplied at 1.8 V have similar dependence at high temperatures (Figure 8 and Figure 9b). At low temperatures, the inaccuracy increases up to +1.5 ℃ (Figure 9b) for less than half the samples. Thus, a mean value of 0.44 ℃, with a standard deviation of 0.71 ℃, for the 20 measured temperature errors is observed in Table 1 in the temperature range of −20 to 125 ℃ at 1.8 V.

At a lower power supply voltage (Figure 9a), the inaccuracy at −20 ℃ increases to 1.56 ℃, while for high temperatures, it varies between +0.75 to +1.3 ℃ in almost all cases. At 1.9 V (Figure 9c), the error reported by most of the measured samples varied by ±1 ℃ in the full temperature range. With regards to the mean and standard deviation of the temperature error reported at 1.7 and 1.9 V, they have similar values to the ones obtained at 1.8 V (Table 1).

The performances of the trimmed thermal sensor (Figure 3) are compared with recently reported digital temperature sensors [4,5,6,7,8,9,10,11,12,13,14,15,16,17,18,19,20,21,22,23,24,25] in Table 2. The experimental results of 25 measured DTS samples are in good agreement with the majority of referred data [4,5,6,11,12,13,14,15,18,19,21,24], providing low inaccuracy in a wide temperature range. Furthermore, the proposed trimmed DTS can be supplied with an extended domain of supply voltages. The presented digital sensor achieves its performance by calibration to just one point, while the inaccuracies obtained in [7,8,9,11,13,18,20,21,22,24] are performed using the two-point calibration method. With regards to power consumption, the investigated sensor reaches an acceptable value in comparison with [11,12,18,22].

## 4. Conclusions

A 0.18-μm CMOS reprogrammable fuse using EEcells is proposed for trimming a digital temperature sensor. The fuse uses EEPROM memory cells, which allow multiple programming cycles by altering the stored data for digital trim codes applied to the smart sensor. By reprogramming the fuse, the digital sensor can be adjusted with an increased trim variation in order to achieve higher accuracy. The operation of the trimmed DTS was validated by Synopsis HSPICE simulations and wafer-level and encapsulated IC measurements. A +1.5/−1.0 °C inaccuracy in the −20 to 125 °C range was obtained for 25 DTS measured samples at 1.8 V by one-point calibration, while the mean was centered at 0.44 °C, with a standard deviation of 0.71 °C. The digital sensor exhibits similar results for a power supply range of 1.7 to 3.6 V. Thus, the DTS’s performance is in fairly good agreement with recently reported temperature sensors, and the proposed trimming technique can be used in multiple presented applications.

## Figures and Tables

**Table 1:** Mean and standard deviation of the temperature error for 20 measured DTS samples.

Parameter	Power Supply [V]	Temperature Range [°C] −20~125
Mean [°C]	1.7	0.30
	1.8	0.44
	1.9	0.35
Standard Deviation [°C]	1.7	0.71
	1.8	0.71
	1.9	0.69

**Table 2:** Comparison with recently reported digital temperature sensors.

	Type	Process [nm]	Power Supply [V]	Temperature Range [°C]	Inaccuracy [°C]	Calibration	No. Samples	Power [µW]
This Work	BJT	180	1.7~3.6	−20~125	+1.56/−1.0	One point	25	850
[4]	TD	55	0.8~1.3	−40~125	±0.70 (3σ)/±0.94 (3σ)	Two points	4	9.3
				−10~110	±1.38 (3σ)/±1.64 (3σ)	One point	4	9.8
[5]	CIS pixels	180	3.3	−20~80	±1.30 (3σ)	Two points	3	36
[6]	CIS pixels	180	3.3	0~100	±1.40 (3σ)	Two points	3	144
[7]	MOS	130	1.3	−20~85	±0.60(3σ)	Two points	10	6
[8]	TD	130	1.3	−20~85	±0.60 (3σ)	Two points	10	6
[9]	MOS	180	1.0	0~100	+0.29/−0.98	Two points	10	22.3
[10]	BJT	160	1.8	−40~180	±0.25 (3σ)	One point	24	9.75
[11]	MOS	350	3.3	0~90	+0.7/−1.35	Two points	3	3000
[12]	BJT	180	-	16~87	+0.68/−0.8	One point	3	586
[13]	RES	65	0.6~1.0	−20~120	±1.5 (3σ)/0.80 (3σ)	Two points	16	0.1
[14]	RES	65	0.6~1.2	−45~85	+1.6/−1.0	Two points	8	47.2
					±4	One point	8	
[15]	RES	65	1.0	0~100	+1.5/−1.1	One point	12	36
[16]	BJT&MOS	22	1.0	−30~120	±1.07 (3σ)	One point	38	50
[17]	TD	180	1.2	−40~120	+1.0/−1.0	One point	4	3
[18]	BJT	14	1.35	0~100	+1.0/−1.5	Two points	52	1600
[19]	MOS	60	1.0	20~80	±3	One point	4	14
[20]	BJT	-	3.0~5.5	−20 ~55	±0.15	Two points	14	-
[21]	MOS	180	0.8	−20 ~80	+1.2/−0.9	Two points	9	11
[22]	BJT	180	1.8	−55 ~125	±0.3 (3σ)	Two points	20	8280
[23]	BJT	180	1.8–5.5	20 ~50	±0.1 (3σ)	One point	15	16
[24]	MOS	180	1.2	0~100	+1.5/−1.4	Two points	4	0.071
[25]	RES	65	0.83–1.35	−50~105	±1.2 (3σ)	One point	20	32.5

## Data Availability

Data sharing not applicable.
